# Supplementary material for: Remote Actuation of Apoptosis in Liver Cancer Cells via Magneto-Mechanical Modulation of Iron Oxide Nanoparticles
Source: Cancers (Basel). 2019 Nov 26;11(12):1873. doi: 10.3390/cancers11121873 (PMC6966689; doi:10.3390/cancers11121873)
Supplement: Supplementary file 1 [file cancers-11-01873-s001.pdf]

Supplemental Materials

Remote Actuation of Apoptosis in Liver Cancer Cells via Magneto-mechanical Modulation of Iron Oxide Nanoparticles

Oleg Lunov [1,*], Mariia Uzhytchak [1], Barbora Smolková [1], Mariia Lunova [1,2], Milan Jirsa [2], Nora M. Dempsey [3], André L. Dias [3], Marlio Bonfim [4], Martin Hof [5], Piotr Jurkiewicz [5], Yuri Petrenko [6], Šárka Kubinová [1,6] and Alexandr Dejneka [1]

[1] Institute of Physics of the Czech Academy of Sciences, Prague, 18221, Czech Republic; lunov@fzu.cz (O.L.); uzhytchak@fzu.cz (M.U.); smolkova@fzu.cz (B.S.); dejneka@fzu.cz (A.D.)

[2] Institute for Clinical & Experimental Medicine (IKEM), Prague, 14021, Czech Republic; mariialunova@googlemail.com (M.L.); miji@ikem.cz (M.J.)

[3] Université Grenoble Alpes, CNRS, Grenoble INP, Institut Néel, 38000 Grenoble, France; nora.dempsey@neel.cnrs.fr (N.M.D.); andre.dias@neel.cnrs.fr (A.L.D.)

[4] Universidade Federal do Paraná, DELT, Curitiba, 81531-980, Brazil; marliob@eletrica.ufpr.br (M.B.)

[5] J. Heyrovský Institute of Physical Chemistry of the Czech Academy of Sciences, Prague, 18223, Czech Republic; martin.hof@jh-inst.cas.cz (M.H.); piotr.jurkiewicz@jh-inst.cas.cz (P.J.)

[6] Institute of Experimental Medicine of the Czech Academy of Sciences, Prague, 14220, Czech Republic; yuriy.petrenko@iem.cas.cz (Y.P.); sarka.kubinova@iem.cas.cz (S.K.)

* Correspondence: lunov@fzu.cz; Tel.: +420266052131 (O.L.)

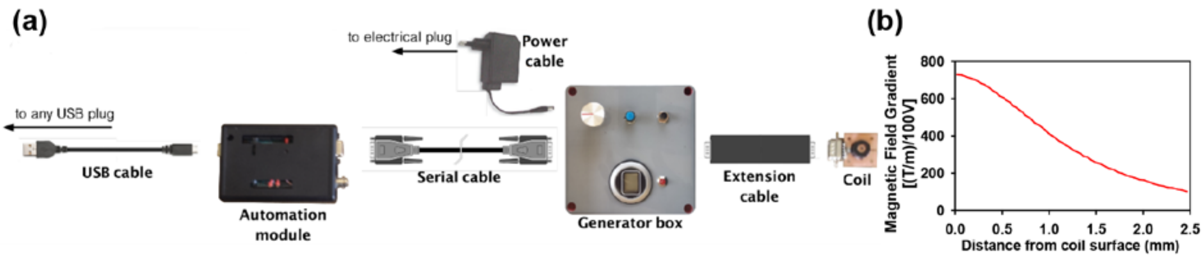

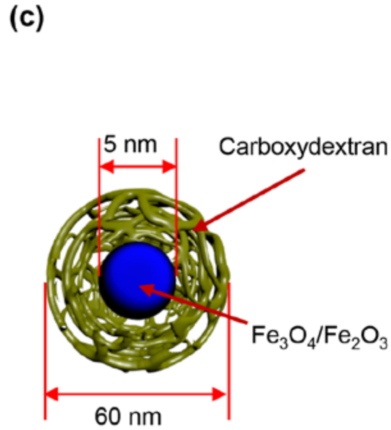

Particle	ξ (mV)	PDI	Mobility (μmcm/V_s)	Hydrodynamic diameter (nm)	Conductivity (mS/cm)
SPION	-12.8	0.211	-1.01	60.6	20.7
SPION-redF	-13.4	0.234	-1.05	64.1	19.5

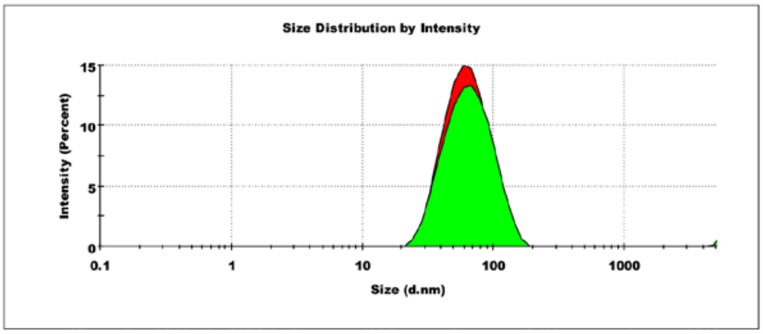

Figure S1 The magnetic pulse generator scheme and characterization of SPIONs. (a) The magnetic pulse generator is a device designed to create magnetic fields of up to 10 T outside the generating coil. The system consists of a pulsed current source coupled with a Cu coil of inner (outer) diameter 3 (7) mm. The current source is very compact (roughly 10 x 10 x 10 cm^3) and as the current pulses are very short (~ 15 μs), there is no need to cool the coil. (b) Simulation of the maximum magnetic field gradient as a function of the distance from the surface of the coil. (c) Surface characterization of the particles dissolved in PBS measured with a Zetasizer Nano (Malvern Instruments). PDI – polydispersity index; ζ – zeta potential.

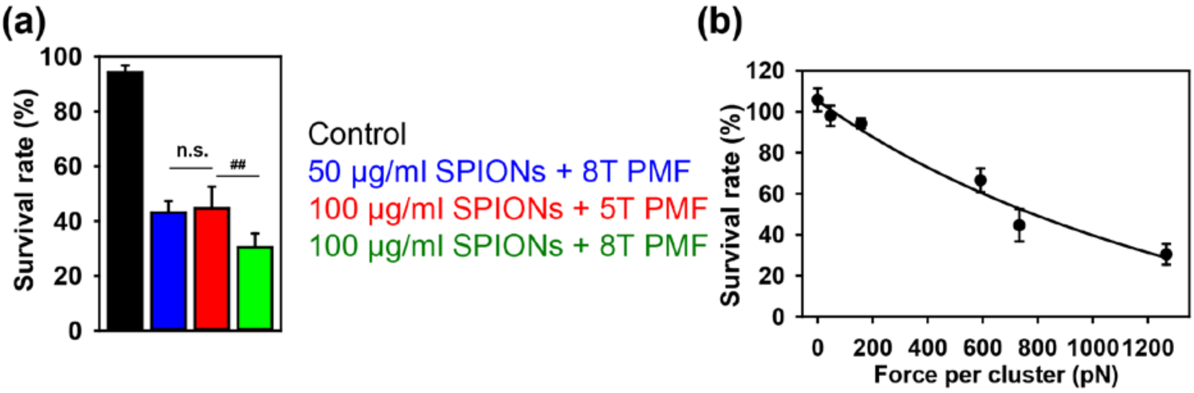

Figure S2 Cell survival rate as a function of the magnetic gradient force exerted on clusters of SPIONs. (a) Huh7 cells were pre-incubated with different concentrations of SPIONs (50, 100 μg Fe mL^{-1}) for 1.5 h. Incubated cells with incorporated nanoparticles were exposed to PMF (10 pulses of either ~ 8 T or ~ 5 T at intervals of 10 sec). 24 h later cell viability was assessed by the WST-1 assay. The data were normalized to control values (no particles, no PMF exposure) and expressed as mean ± SEM, n = 3 each. ##*p* < 0.01 denotes significant

differences. (b) Huh7 survival rate as a function of the magnetic gradient force exerted on clusters of SPIONs.

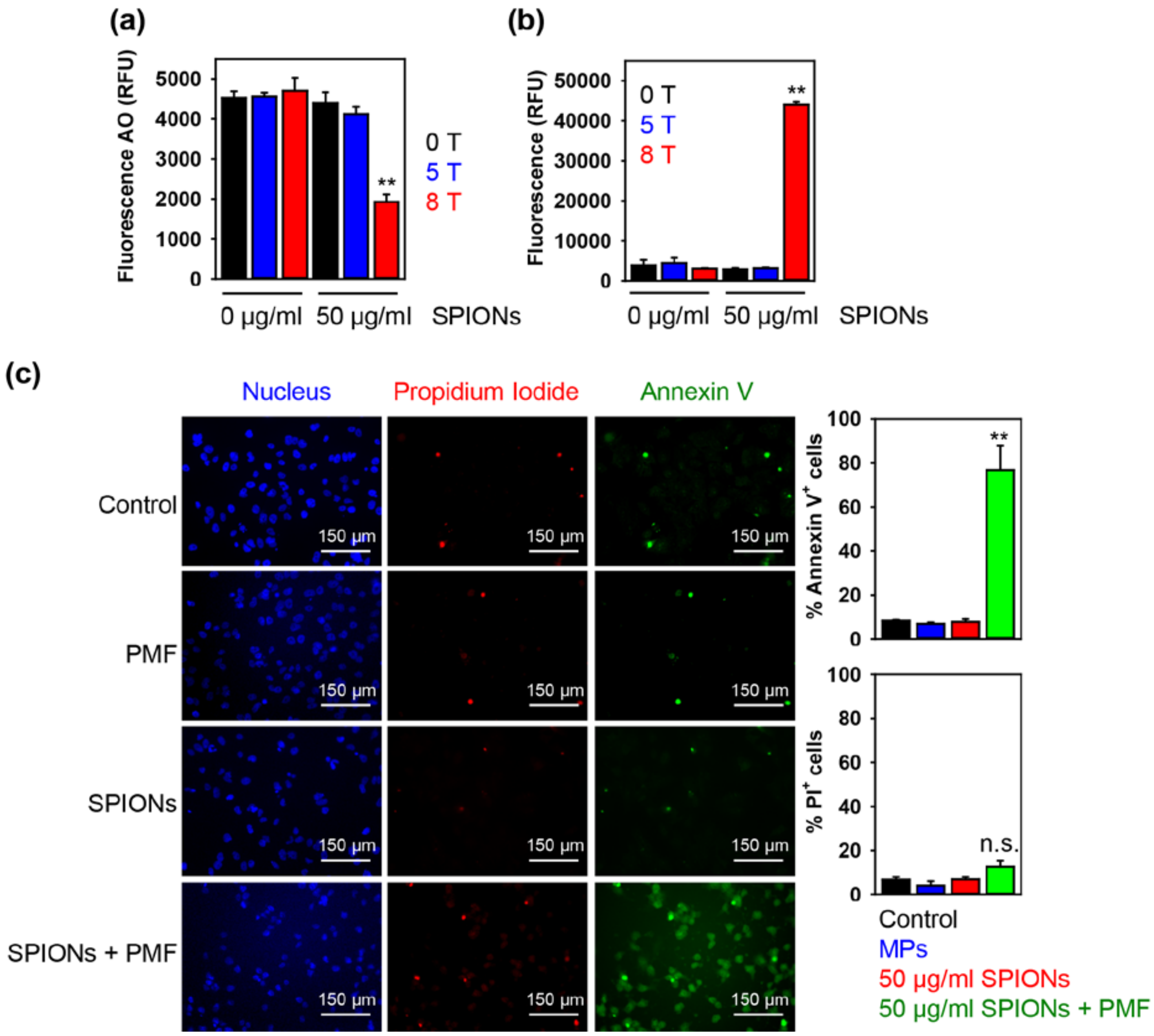

Figure S3 Apoptosis assessment in cells exposed to PMF (10 pulses of ~ 8 T at intervals of 10 sec). (a) Huh7 cells were pre-incubated with SPIONs 50 µg Fe mL^{-1} for 1.5 h. After incubation cells with incorporated nanoparticles were exposed to PMF (10 pulses of either ~ 8 T or ~ 5 T at intervals of 10 sec), stained with acridine orange (AO) and then, orange fluorescence intensity was measured using a fluorescent microplate reader (Tecan Infinite® 200 PRO). The data is expressed as mean ± SEM, n = 3 each. $**p < 0.01$ denote significant differences respect to control (no particle, no PMF treatment). (b) Huh7 cells were treated as in (a). After PMF treatment caspase-3 activity was assessed using a ApoStat detection kit (R&D Systems) and analyzed by fluorescent microplate reader (Tecan Infinite® 200 PRO). The data are expressed as mean ± SEM, n = 3 each. $**p < 0.01$ denote significant differences with respect to control (no particles, no PMF treatment). (c) Huh7 cells were pre-incubated with SPIONs 50 µg Fe mL^{-1} for 1.5 h. After cells with incorporated nanoparticles were exposed to PMF (10 pulses of ~ 8 T at intervals of 10 sec), then 4 h after treatment cells were labelled with Hoechst nuclear stain – blue dye, annexin V – green dye and propidium iodide – red dye. Labelled cells were imaged with fluorescence microscopy. Representative images out of three independent experiments are shown. Quantification of annexin V and PI was assessed using ImageJ

software (NIH). The data is expressed as mean ± SEM, n = 3 each. **p < 0.01 denotes significant differences respect to control (no particles, no PMF treatment).

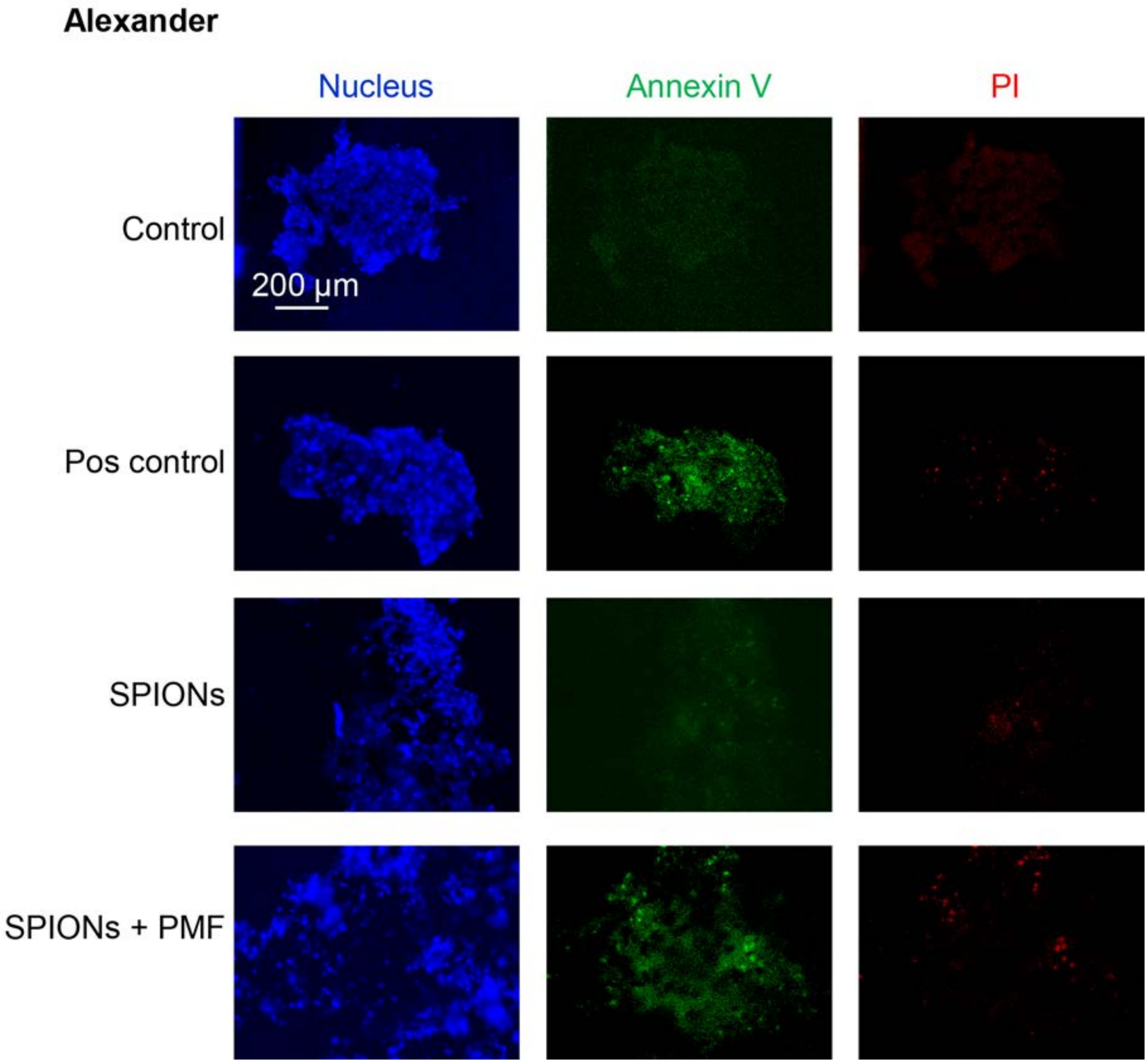

Figure S4 Organoid-like 3D structures from Alexander cells were treated for 2 h with SPIONs 100 µg Fe mL^{-1}. After cells with incorporated nanoparticles were exposed to PMF (10 pulses of ~ 8 T at intervals of 10 sec), then 6 h after treatment cells were labelled with Hoechst nuclear stain – blue dye, annexin V – green dye and propidium iodide – red dye. Labelled cells were imaged with fluorescent microscopy. Representative images out of three independent experiments are shown. Positive control – 2 µM staurosporine for 6 h.

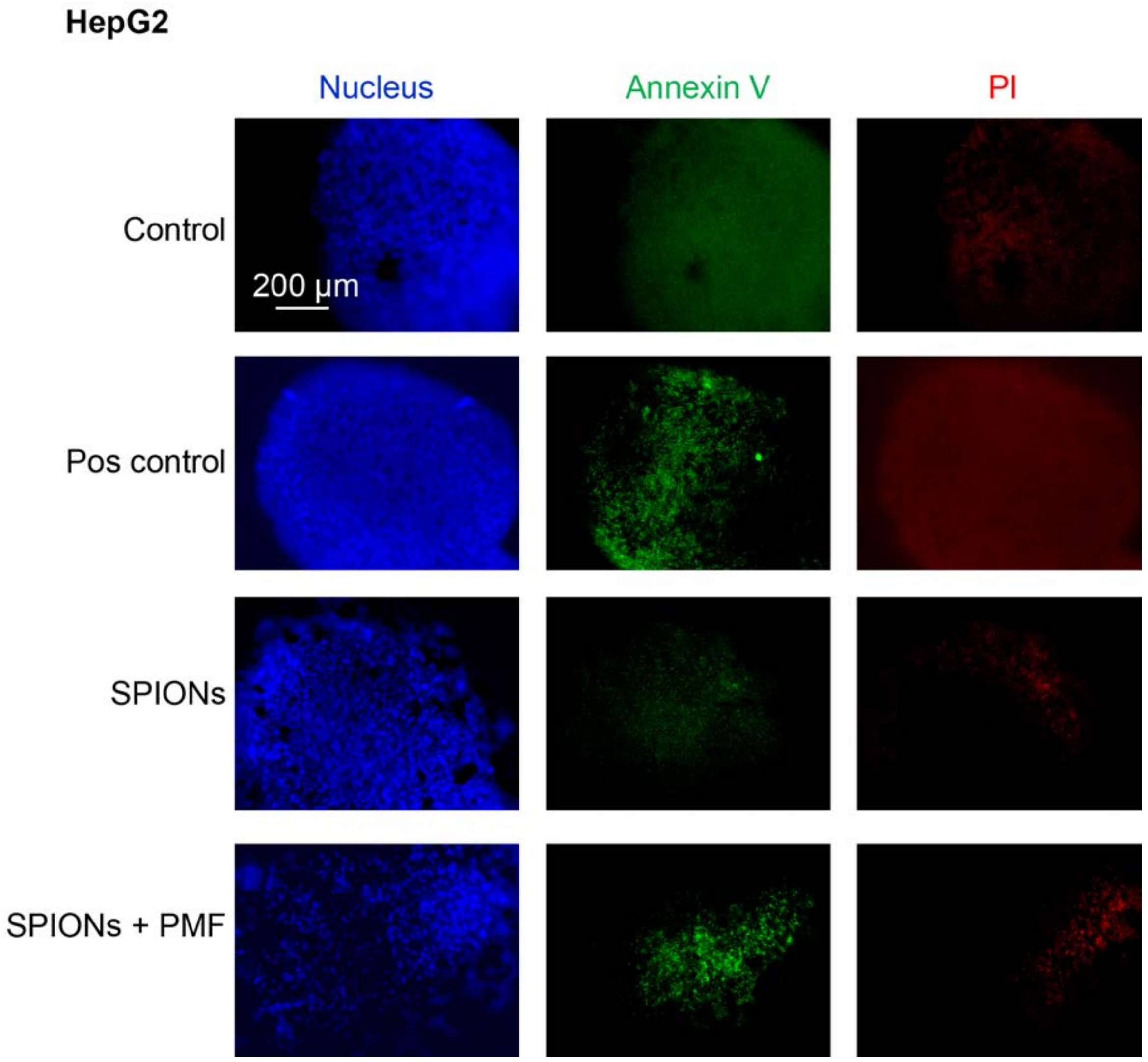

Figure S5 Organoid-like 3D structures from HepG2 cells were treated for 2 h with SPIONs 100 µg Fe mL^{-1}. After cells with incorporated nanoparticles were exposed to PMF (10 pulses of ~ 8 T at intervals of 10 sec), then 6 h after treatment cells were labelled with Hoechst nuclear stain – blue dye, annexin V – green dye and propidium iodide – red dye. Labelled cells were imaged with fluorescent microscopy. Representative images out of three independent experiments are shown. Positive control – 2 µM staurosporine for 6 h.

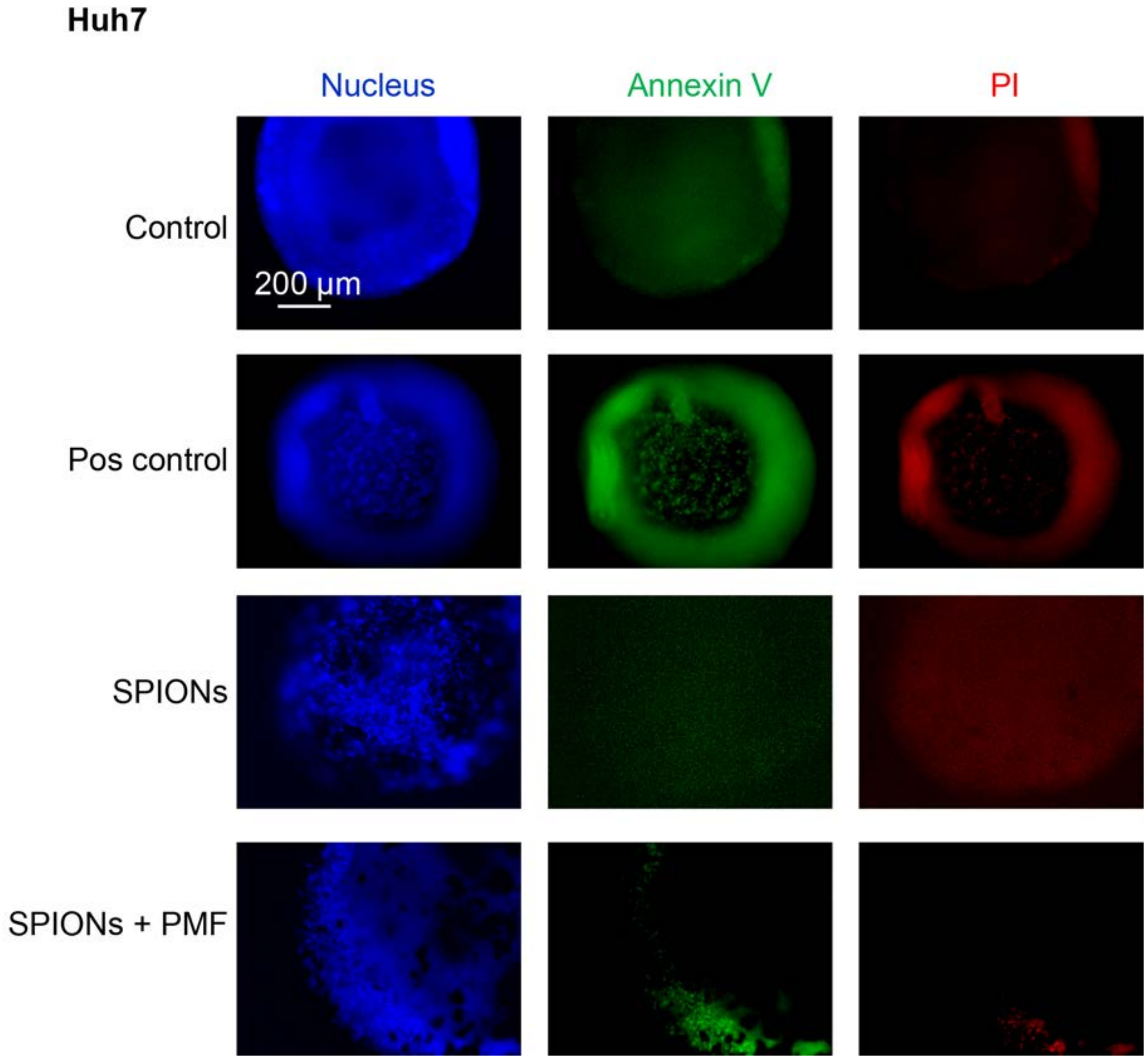

Figure S6 Organoid-like 3D structures from Huh7 cells were treated for 2 h with SPIONs 100 μg Fe mL^{-1}. After cells with incorporated nanoparticles were exposed to PMF (10 pulses of ~ 8 T at intervals of 10 sec), then 6 h after treatment cells were labelled with Hoechst nuclear stain – blue dye, annexin V – green dye and propidium iodide – red dye. Labelled cells were imaged with fluorescent microscopy. Representative images out of three independent experiments are shown. Positive control – 2 μM staurosporine for 6 h.
